# Rapidly dissolving polymeric microneedles for minimally invasive intraocular drug delivery

**DOI:** 10.1007/s13346-016-0332-9

**Published:** 2016-10-05

**Authors:** Raghu Raj Singh Thakur, Ismaiel A. Tekko, Farhan Al-Shammari, Ahlam A. Ali, Helen McCarthy, Ryan F. Donnelly

**Affiliations:** 1 School of Pharmacy, Medical Biology Centre, Queen’s University Belfast, 97 Lisburn Road, Belfast, BT9 7BL Northern Ireland UK; 2 Faculty of Pharmacy, Aleppo University, Aleppo, Syria

**Keywords:** Microneedles, Ocular drug delivery, FITC-dextran, Cornea, Sclera, Polyvinylpyrrolidone (PVP)

## Abstract

In this study, dissolving microneedles (MNs) were used to enhance ocular drug delivery of macromolecules. MNs were fabricated using polyvinylpyrrolidone (PVP) polymer of various molecular weights (MWs) containing three model molecules of increasing MW, namely fluorescein sodium and fluorescein isothiocyanate–dextrans (with MW of 70 k and 150 k Da). Arrays (3 × 3) of PVP MNs with conical shape measuring about 800 μm in height with a 300 μm base diameter, containing the model drugs, were fabricated and characterized for their fracture forces, insertion forces (in the sclera and cornea), depth of penetration (using OCT and confocal imaging), dissolution time and in vitro permeation. The average drug content of the MNs (only in MN shafts) ranged from 0.96 to 9.91 μg, and the average moisture content was below 11 %. High MW PVP produced MNs that can withstand higher forces with minimal reduction in needle height. PVP MNs showed rapid dissolution that ranged from 10 to 180 s, which was dependent upon PVP’s MW. In vitro studies showed significant enhancement of macromolecule permeation when MNs were used, across both the corneal and scleral tissues, in comparison to topically applied aqueous solutions. Confocal images showed that the macromolecules formed depots within the tissues, which led to sustained permeation. However, use of MNs did not significantly benefit the permeation of small molecules; nevertheless, MN application has the potential for drug retention within the selected ocular tissues unlike topical application for small molecules. The material used in the fabrication of the MNs was found to be biocompatible with retinal cells (i.e. ARPE-19). Overall, this study reported the design and fabrication of minimally invasive rapidly dissolving polymeric MN arrays which were able to deliver high MW molecules to the eye via the intrastromal or intrascleral route. Thus, dissolving MNs have potential applications in enhancing ocular delivery of both small and macromolecules.

## Introduction

Ocular diseases such as age-related macular degeneration (AMD), diabetic retinopathy, posterior uveitis and retinitis due to glaucoma that affects the posterior segment of the eye are among the leading causes of vision loss worldwide [1–3]. Other diseases such as fungal keratitis and corneal neovascularization (CNV) that affect the anterior segment of the eye can also cause visual impairment. For example, CNV is considered a serious corneal ailment that affects more than 1.4 million people in the USA and has lead to vision loss in 12 % of them [4, 5].

Fortunately, rapid expansions of new technologies in ocular drug delivery and development of new drug candidates, including biologics, to combat these challenging diseases have recently emerged [6]. However, an effective drug delivery system that can selectively localize the drug within the target ocular tissue (e.g. the sclera or cornea) in sufficient amounts and sustain its release is still challenging for treating ocular diseases.

The most common ocular drug delivery routes include topical, systemic or local administration. However, each route has its own limitations. Although topical administration is the highly preferred route to treat anterior segment diseases, the ocular bioavailability of topically applied drugs is less than 5 % and often less than 1 %. Thus, it is difficult to achieve therapeutic drug concentrations at the target site, for example, in treating CNV and AMD. The poor ocular bioavailability after topical application mainly results from the precorneal factors such as blinking, transient residence time in the cul-de-sac and nasolacrimal drainage. In addition, the lipoidal nature of the corneal epithelium restricts the entry of hydrophilic drug molecules, and the water-laden stroma acts as a rate limiting membrane for lipophilic molecules [7]. Moreover, the physicochemical properties of the drug molecule determine the diffusion resistance and relative impermeability offered by various ocular tissues [8, 9]. Alternatively, systemic or local routes have been used. Unfortunately, systemic administration is not very effective due to the several ocular-blood barriers. Thus, managing ocular disease often requires higher dosage and frequent administration that may result in severe adverse effects [10].

On the other hand, intraocular injections, such as intravitreal and subconjunctival injections, have been widely used as alternate strategies to achieve therapeutic drug concentrations in treating ocular disorders. Particularly, intravitreal injections, using conventional hypodermic needles, of macromolecules such as anti**-**vascular endothelial growth factors (anti-VEGF) ranibizumab (Lucentis®, 48 k Da), bevacizumab (Avastin®, 149 k Da) and aflibercept (Eylea®, 97 kDa) are commonly practiced to treat posterior segment diseases (AMD) [11]. However, to maintain effective concentrations of these drugs repeated injections are required, which causes discomfort to patients and is associated with serious clinical complications such as vitreous hemorrhage, retinal detachment, or endophthalmitis [12]. Thus, an effective ocular drug delivery system with minimal side effects is still lacking, and the great demands for such an effective drug delivery system formed the driving force for a very active research among pharmaceutical and biomedical scientists.

In attempt to improve ophthalmic drug delivery, several alternate approaches have been proposed and investigated including use of microneedles (MNs). MNs are an attractive technology in micron scale that is minimally invasive and has been extensively investigated over the last 15 years to enhance transdermal drug and gene delivery and monitoring [13–15]. To date, various types of MNs have been fabricated and tested including solid, hollow and dissolving polymeric MNs [16–18]. Recently, attempts have been made to use the solid and hollow MNs for ocular drug delivery. Drug-coated solid MNs as well as hollow MNs made from a variety of materials have been investigated to deliver model drugs of various molecular weights (MWs), and nano- or microparticles into the eye either via intracorneal or intrascleral routes [19–22]. While various designs of MNs have been shown to be effective in improving ocular drug delivery, some drawbacks may impose limitations on device performance that ultimately constrain their applicability and efficacy. The main drawbacks were lack of accuracy and reproducibility for drug-coated solid MNs [19], and brittleness, complication and difficulty of fabrication and drug infusion into the ocular tissues using silicon-made hollow and solid MNs [20, 21]. For further information, readers are directed to read a recent review on ocular application of MNs [23].

Interestingly, dissolving polymeric MNs have shown a promising strategy for transdermal gene and drug delivery, yet no data have been reported concerning their feasibility for ocular drug delivery. Using dissolving MNs could avoid the above-mentioned drawbacks associated with solid and hollow MNs and minimize the solid materials burden and the potential accidental retinal damage and detachment upon using solid and hollow MNs. Essentially, dissolving MNs will soften and dissolve within the ocular tissues upon penetration preventing highly sensitive tissues such as the retina from damage due to mechanical forces of application.

In this study, we have investigated the potential of using dissolving MN arrays prepared from FDA-approved biocompatible polymers. In this regard, we have used polyvinylpyrrolidone (PVP) which is an FDA-approved biocompatible polymer and has been widely used as a blood plasma expander and in pharmaceutical industry for several purposes including as a tablet binder [24] and in fabricating dissolving MNs for transdermal drug delivery [25–27]. We hypothesize that we can use a simple and cost-effective method in fabrication of rapidly dissolving MNs using biocompatible polymers that can successfully encapsulate drugs of various MWs and to be strong enough to penetrate the ocular tissues, and rapidly dissolve to deliver the payload to both anterior and posterior segments of the eye in a minimally invasive manner. To evaluate our hypothesis, we have used a mould-casting method in the fabrication of a series of PVP-based dissolving MN arrays containing representative model drugs that are commonly employed in the treatment of eye diseases. MNs were loaded with either low MW fluorescein sodium (FS, 376.27 Da) or a model macromolecule i.e. fluorescein isothiocyanate (FITC) labeled dextran of 70 kDa (FD 70) or 150 kDa (FD 150), respectively, incorporated into the PVP gels at different concentrations. The feasibility of fabricating PVP-based MN arrays was evaluated using PVP of various MWs i.e. PVP K15 (MW 10 k Da), PVP K30 (MW 40 k Da) or PVP K29–32 (58 k Da). The fabricated MN arrays were investigated in terms of their mechanical strength, their ability to penetrate the corneal and scleral tissues, dissolution kinetics, drug and moisture content. Furthermore, the ability to deliver the model drugs into the anterior and posterior eye segments via intracorneal and intrascleral application was evaluated in an in vitro study. Drug distribution within the selected ocular tissues was also determined, and biocompatibility of the MNs was evaluated in an in vitro study using human retinal epithelium cell lines.

## Materials and methods

### Materials

Fluorescein isothiocyanate–dextran with an average MW of 70,000 Da (FD 70) and 150,000 Da) were purchased from TdB Consultancy AB (Uppsala, Sweden). Fluorescein sodium (FS) MW 376.27 Da and polyvinylpyrrolidone (PVP K15, MW 10,000 Da; PVP K30, MW 40,000 Da; PVP K90, MW 360,000 Da) were all purchased from Sigma–Aldrich (Dorset, UK). Polyvinylpyrrolidone (PVP K29–32, MW 58,000 Da) was a gift from Ashland Inc. (Surrey, UK). ARPE-19 cells were purchased from the American Type Culture Collection. Dulbecco’s modified Eagle’s medium/F-12 human amniotic membrane nutrient mixture (DMEM) was purchased from (Life technologies, UK). Fetal bovine serum (FBS) and trypsin were purchased from Invitrogen Corporation (Paisley, UK). The 3-(4,5-dimethyl-2-thiazolyl)-2,5-diphenyl-2H-tetrazolium bromide (MTT) cell viability reagent was purchased from Sigma Aldrich (Dorset, UK). All other chemicals used were of analytical reagent grade.

### Drug solutions preparation

A 10 mg of FS, FD70 or FD150 were each dissolved in a known volume of deionized water and then adjusted to 5 ml. The solutions were left for 5 h under constant stirring of 400 rpm at room temperature and stored in a plastic caped glass bottle covered with aluminium foil at room temperature until further use.

### Preparation of drug-loaded PVP hydrogels

PVP hydrogels were prepared in double distilled water according to the composition presented in Table 1. A predefined amount of polymer was added to a known volume of deionized water and then vortexed for 60 s. The suspension was then sonicated at 37 °C for 60 min and stored for overnight hydration at room temperature. The volume of hydrogel was then adjusted by adding the reminder of deionized water to make the required polymer concentration and mixed vigorously for homogenization forming the PVP hydrogels. These gels were used to prepare drug-containing hydrogel formulations. For this, a predetermined amount of each model drug was added to a specified PVP hydrogel to provide a final drug concentration of either 2 or 10 mg/g, as presented in Table 1, and then homogeneously dispersed for 5 h using magnetic stirring operated at 400 rpm at room temperature. The PVP/drug hydrogel formulations were kept in cap-closed glass container wrapped in aluminium foil at 4–8 °C until further use. For MN baseplates, in all cases, 15 % *w*/*w* of PVP K90 hydrogels were used, as this formed mechanically strong back support (i.e. baseplates) to the MN arrays due to its high MW, while lower MW PVP hydrogels produced very brittle baseplates.

**Table 1:** Composition of PVP-based MNs

Formulation code	PVP type and concentration (%, *w*/*w*)	Model drug	Drug concentration in hydrogel (mg/g)
P1	PVP K29–32/30	–	–
P2	PVP K30/40	–	–
P3	PVP K15/60	–	–
F1	PVP K29–32/30	FS	2
F2	PVP K29–32/30	FD70	2
F3	PVP K29–32/30	FD150	2
F4	PVP K29–32/30	FD70	10
F5	PVP K15/60	FD70	2
F6	PVP K30/40	FD70	2

### Microneedles fabrication

Laser-engineered silicone micromould templates composed of 9 (3 × 3) conical shaped needles, with an average height of 800 μm, a base width of 300 μm and an interspacing of 50 μm, were used for microfabrication of MN arrays. The method of mould fabrication is detailed in our previous publication [28]. Briefly, approximately 100 mg of hydrogel formulation of PVP (without drug) or PVP (with drug) was poured into the silicone moulds and centrifuged for 5 min at 5000×*g* to form the MNs shafts alone. The needles were left to settle and from a highly viscous gel under gentle airflow from an electric fan for about 30 min to allow partial drying. This was followed by adding approx. 400 mg of 15 % *w*/*w* PVP K90 gel and centrifuged again for 5 min at 5000×*g* to form the MN baseplate. This method of fabrication reduces the issue of drug migration from MN arrays to the baseplate. Following centrifugation, the MN arrays were dried in the moulds at room temperature for 48 h. The MN arrays were then carefully removed from the molds and assessed visually for mechanical strength and formulation homogeneity and then kept in a container sealed with aluminium foil until use.

### Preparation of ocular tissues

Ocular tissues were prepared from porcine eyeballs. Porcine eyes have been widely used as a good model for human eye for in vitro permeation studies. Importantly, because they have a similar histology, collagen bundle organization and water content to that of the human eye, except for the sclera, which is a twofold thicker [29, 30], porcine eyeballs for our studies were procured from a local slaughterhouse. Eyeballs were either used within 24 h of extraction or frozen at −80 °C until further use. After thawing, the adherent muscle tissue was removed from the eye bulb, and the anterior segment of the eye was circumferentially cut behind the limbus. The eye was then cut into two halves; the vitreous humor was removed, and both the anterior sclera and cornea tissues were then isolated by removal of the underling tissues using a cotton swab, soaked in phosphate buffer saline (at pH 7.4) for 30 min and then blotted dry and stored in sealed container at −80 °C until further use. The frozen tissues were used within 3 months.

### Microneedles characterization

All fabricated MNs were characterized for a series of tests namely for drug content, percentage moisture content, mechanical strength (fracture force, insertion force and depth of penetration) and dissolution. Details of each method are provided below:

#### Drug content determination

MN arrays were carefully removed from the baseplates, under a magnifying lens, using scalpel and then dissolved in 5 ml of PBS at room temperature using a magnetic stirring bar operated at 400 rpm for 30 min. An aliquot of 150 μL was then removed and analyzed using a fluorescence plate reader as described below. Data was reported as drug weight (in μg) per array (mean ± SD, *n* = 3).

#### Moisture content determination

The percentage moisture content of the fabricated MN arrays was determined using a Q500 Thermo Gravimetric Analyser (TA Instruments, Elstree, Herts, UK). Samples of 5–10 mg of MN arrays were heated from ambient temperature to 150 °C at a heating rate of 10 °C/min. Nitrogen flow rates of 40 ml/min (balance purge gas) and 60 ml/min (sample purge gas) were maintained for all samples. The data from thermogravimetric analysis experiments was analyzed with TA Instruments Universal Analysis 2000 software, version 4.4 A (TA Instruments, Elstree, Herts, UK). Moisture content was determined from calculating the percentage of reduction in sample weight at 110 °C. Data reported as mean ± SD, *n* = 3.

#### Determination of fracture force

Mechanical strength of dissolving MN arrays was determined by employing a series of predetermined forces. A TA-XT2 Texture Analyser (Stable Microsystems, Haselmere, UK) in compression mode was also used for this test as described previously [27]. Briefly, MN arrays were visualized before testing using a Leica MZ6 dissection microscope (Leica Microsystems UK Ltd., Milton Keynes, UK). Then, MNs array were carefully placed on the flat stainless steel baseplate of the Texture Analyzer probe with the needles pointing downwards. The probe was then lowered at a speed of 0.1 mm/s until MNs touched a smooth stainless steel solid block and then it exerts its predefined force. Upon MNs solid block contact, the probe maintained the designated force for 30 s; after which, it was moved upwards at a speed of 1 mm/s. MN arrays were then visualized using the light microscope, and the MN height was measured. The MN height displacement (mm) was then calculated and reported as the percentage of reduction in the MN height vs applied force. Experiments were run in triplicates.

#### Determination of the insertion force in ocular tissues

The insertion force of MNs into the ocular tissues was determined using a TA-XT2 Texture Analyser (Stable Microsystems, Haslemere, UK) as described previously [27], with minor modifications. The scleral or corneal tissues were hydrated for 2 h in PBS (pH 7.4) incubated in a water bath at 37 °C. Tissues were then mounted on a wooden ball (to represent eyeball shape) fixed underneath the Texture Analyser probe using a metal holder. MN arrays were attached to the probe using double-sided tape (3 M, Carrickmines, Ireland). The Texture Analyzer was set in compression mode, and the trigger force was set to 0.1 N. The probe then moved downwards at a speed of 0.1 mm/s. Upon contact with the scleral/corneal tissue, the probe continued to travel at the same speed until the required insertion depth (0.8 mm) was reached while the compression force was being recorded. Once the target depth was reached, the probe was then moved upwards at a speed of 1 mm/s. Data was presented as insertion force per array vs insertion depth. Experiments were run in triplicates.

#### Determination of depth of penetration in ocular tissues

Further studies were also performed to visualize and measure the MNs depth of penetration in the ocular tissues using optical coherence tomography (OCT) (EX1301 OCT microscope, Michelson Diagnostics, Kent, UK) as described previously [28], with minor modifications. Briefly, fresh ocular tissues were hydrated for 2 h in PBS (pH 7.4) incubated in a water bath at 37 °C and then blotted dry. Hydrated tissues were mounted on a dental wax board. Taking into consideration that MN are rapidly dissolving which will hinder their visualization by OCT, another layer of Parafilm® was used to cover the ocular tissue to prevent MN dissolution upon insertion. MN arrays were then inserted using a custom-made applicator device and visualized immediately using an EX1301 OCT Microscope. The swept-source Fourier domain OCT system has a laser centre wavelength of 1305.0 ± 15.0 nm, facilitating real-time high-resolution imaging of the upper ocular layers (7.5 μm lateral and 10.0 μm vertical resolution). The ocular tissue was scanned at a frame rate of up to 15 B-scans (2D cross-sectional scans) per second (scan width = 2.0 mm). The 2D images were analyzed using the imaging software ImageJ® (National Institutes of Health, Bethesda, USA). The scale of the image files obtained was 1.0 pixel = 4.2 μm, thus allowing accurate measurements of the depth of MN penetration. Three replicates were performed, and the insertion depths of 3 × 3 MN arrays were measured in total.

#### Dissolution of MN arrays

The dissolution of the PVP-based MN arrays with and without model drugs was investigated in situ in both the corneal and scleral tissues. The eye tissues were carefully cut into square pieces using a disposable scalpel then hydrated in PBS (pH 7.4) at a temperature of 37 °C for 2 h. The ocular tissue specimen of either the corneal or the scleral tissues were blotted dry and carefully secured to a custom-made circular sheet from a weighing pan using cynoacrylate glue (Loctite Ltd., Dublin, Ireland). These were then placed on a dental wax board to support the eye tissue, and then MN arrays were inserted into the centre of the tissue using a custom-made applicator device and held there for predefined time periods (0, 10, 20, 60, 120, 180 and 300 s). At each time point, MNs were removed from the eye tissue, flash frozen in liquid nitrogen and stored at −20 °C until viewing. MN arrays were then visualized using a Leica MZ6 dissection microscope (Leica Microsystems UK Ltd., Milton Keynes, UK) fitted with a Nikon Coolpix 950 digital camera (Nikon UK Ltd., Surrey, UK). MN heights were measured before and after insertion. The percentage of reduction in MN height was calculated and reported as MN height remaining vs time (sec). Experiments were conducted in triplicates.

### In vitro intraocular drug distribution studies

Drug distribution within the ocular tissues (i.e. the cornea and sclera) was evaluated using two model drugs i.e. FS as a representative for a small MW drug and FD70 as a representative for a high MW drug. Ocular tissues were prepared as described earlier, and permeation studies were performed as below. An aluminium foil sheet was used to separate the MN baseplate from direct contact with the ocular tissues. Thus, distributed drug comes only from the inserted MN arrays. Either model drug solution or MN arrays were applied for predefined time periods and were removed from the tissue surface. Ocular tissues were then rinsed with fresh PBS solution, blotted dry and placed into sample blocks containing freezing agent (OCT, Sakura Finetechnical, Tokyo, Japan) and snap-frozen with liquid nitrogen. Each tissue sample was sectioned into 50-μm thick pieces using a cryostat microtome (Richard Allan Scientific, Kalmazoo, MI, USA) and collected into consecutive sections onto glass slides. Tissue sections were examined by confocal laser scanning microscope (Leica TCS SP2 Confocal Microscope, Leica Microsystems, UK) fitted with a Nikon Coolpix 950 digital camera (Nikon UK Ltd., Surrey, UK). Z-stacks of a confocal images were rendered into 3D mode using Volocity software (PerkinElmer, UK) to visualize the MN penetration pathways and the nature of drug distribution within the ocular tissues after applying either solution or MN arrays.

### In vitro permeation studies

In vitro permeation (i.e. transscleral, intrascleral, transcorneal and intracorneal) studies were performed using modified Franz-type diffusion cells with an effective diffusional area of (0.6 cm^2^) and a receptor compartment filled with 5 ml PBS (pH 7.4) and stirred with magnetic bar and thermostated to 37 ± 1 °C. Either scleral or corneal tissues were mounted on Franz-type diffusion cells and hydrated for 2 h. A volume of 10 μL of FS, 50 μL of FD 70 or FD 150 aqueous solution, at a concentration of 2 mg/ml, or a single drug-loaded MN array was inserted into the tissues using a custom-made applicator. While the model drug containing MN arrays were removed 5 min after application, drug aqueous solutions were kept in the donor compartment during the course of the permeation study. The baseplate was separated from the corneal/scleral tissues using aluminium foil to ensure that the permeated model drug comes solely from the dissolving MN arrays. The donor compartments and sampling arms were sealed using Parafilm®. Syringes (1.0 ml) with 8.0 cm long and 20G stainless steel needles were used to remove 150 μL from the receptor compartment of the diffusion cells at predetermined time points, which was replaced with an equal volume of prewarmed PBS. Samples were kept in the fridge for further analysis using a fluorescence plate reader. Percentage cumulative permeation of each drug model through the scleral/corneal tissue was determined. Results were reported as mean ± SD, *n* = 3.

### Analytical methods

Firstly, a series of dilutions (at least six dilutions) of each model drug i.e. FS, FD 70, and FD 150 was prepared in PBS. The fluorescent emission of an aliquot of 150 μL sample of each drug solution was measured using a fluorescent plate reader (BMG FLUOstar OPTIMA Microplate Reader, BMG Labtech, Ortenberg, Germany) operated at 37 °C using 493 and 520 nm as excitation and emission wavelengths, respectively. For drug analysis, the gain was set at 1000 and 1500 for FS and FD 70/150, respectively. Standard calibration curves of fluorescence absorbance vs concentration (μg/ml) were constructed and used to calculate drug concentrations in the analyzed samples.

### Biocompatibility studies

As the polymeric material and drug are deposited within the ocular tissue following MN application, evaluating the polymers compatibility with the selected ocular tissues will be a key requirement of developing successful MN formulation. Therefore, PVP’s ocular cells biocompatibility study was performed on human retinal pigment epithelial (ARPE-19) cells. For this, PVP K29/32 was dissolved in cell culture medium at various concentrations (1, 2, 3 and 4 mg/ml) and was filter-sterilized using a 0.2-μm filter. ARPE-19 cells were seeded at a density of 20,000 cells per well onto 96-well tissue culture plates (VWR, Leicestershire, England, UK) for 24 h prior to the assay. On the following day, 200 μL cell culture medium was removed and replaced with 200 μL of PVP K29/32 solutions at different concentrations. Cells were then returned to the incubator for 24 h. The cells were viewed and images were recorded using an inverted microscope at predefined times 0, 2, 4, 6 and 24 h after polymer solution application. To assess cell viability, cell monolayers were washed with 200 μL sterile PBS (pH 7.4) and were then replenished with MEM-containing MTT (3-(4, 5-dimethylthiazol-2-yl)-2,5-diphenyltetrazolium bromide) reagent at 1 mg/ml. The cells were then returned to the incubator for a final 2 h. The MTT assay is a gold standard assay for determining cell viability and is based on the ability of viable cells to reduce the water-soluble MTT to a water-insoluble formazan product. The plates were shaken for 2 min to assist solubilization, before 200 μL aliquots were transferred to duplicate wells of a 96-well microplate for absorbance measurement. The absorbance was measured at 450 nm. The measured absorbance values were expressed as a percentage of the control untreated cells, defined as 100 % viable. The experiment was repeated on three consecutive days. Data was reported as mean ± SD, *n* = 3.

### Statistical analysis

Where appropriate, data was analyzed using the Student *t* test, one-way ANOVA with post-hoc comparisons or Mann–Whitney U-test. In all cases, statistical significance was defined at the standard 5 % level. Microsoft Excel 11.0 (Microsoft) and GraphPad Prism Version 4.0 (GraphPad Prism Software Inc., San Diego, CA, USA) were used to analyze data.

## Results and discussion

Recently, drug-coated solid as well as hollow MNs made from a variety of materials have been investigated to deliver model drugs of various MWs, and nano- or microparticles into the eye either via intracorneal or intrascleral routes [19–21, 22]. Drug-coated stainless steel solid MN was able to deliver only 69 % of the applied dose. The rest of the dose either remained adherent to the MN which was likely due to the incomplete MN insertion into the tissue or may have deposited on the scleral surface affecting dose accuracy and reproducibility [19]. Solid silicon or hollow glass-based MNs are intrinsically brittle and can be broken off accidently during application (thereby remaining in the tissue), and fabrication of these needles is rather complicated and expensive. Furthermore, hollow MNs cannot deliver solutions without MN retraction or use of tissue dissolving enzymes to provide free space for accommodating drug solution that requires careful infusion at a predetermined rate. Drug solution infusion through MNs also requires operation at high pressures (250–300 kPa), which in turn is related to the viscosity of the solution and the geometric properties of the MNs [20, 21]. Uncontrolled retraction from the sclera could lead to removal of the MN and leakage of the drug onto the scleral/corneal surface affecting the amount of drug delivered. Thus, special insertion devices and infusion systems are required to enable MN injection in a controlled manner. Consequently, there is a distinct need for the development of other types of MNs to address these shortcomings. Therefore, this study used PVP-based dissolving polymeric MNs.

A wide range of polymers has been used to fabricate dissolving MN arrays including polyvinylpyrrolidone (PVP) [31]. PVP is a homopolymer formed by the monomer N-vinyl-2-pyrrolidone. It is an FDA-approved biocompatible polymer and has been widely used as a blood plasma expander and in pharmaceutical industry for several purposes including as a tablet binder [24] and in fabricating dissolving MNs for transdermal drug delivery [25–27]. Its wide use can be attributed to a number of properties, including excellent water solubility, biological compatibility, low toxicity, film forming and adhesion, complexing ability, inert behavior towards salts and acids and resistance to thermal degradation in solution [32]. One of the enticing properties of using PVP as the structural material for polymeric MNs is the ability for rapid dissolution within the biological tissues due to the high water solubility, which is deemed essential for ocular drug delivery as it is very challenging to apply the MNs patch on the eye for long periods of time. FS and FITC-conjugated dextrans have been widely used as model molecules in investigations of permeation across ocular tissues, and it was deemed to be stable and reliable tracers [33–35]. Therefore, we have used these molecules in our study as model compounds.

### Microneedles characterization

**Fig. 1:**
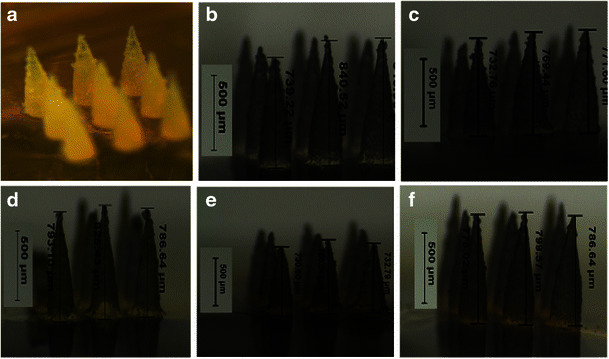
Light microscopy images of MN arrays prepared by using various PVP/drug hydrogel formulations: **a** F1, **b** F2, **c** F3, **d** F4, **e** F5 and **f** F6

In literature, some studies have reported using PVP to prepare dissolving MN arrays e.g. PVP K29/32 hydrogel at 40 % *w*/*v* [27] or 50 % *w*/*v* of either PVP K15 or PVP K29/32 hydrogel [36]. In this study, PVP MN arrays prepared from various MWs were found to be sufficiently rigid from all tested PVP hydrogel formulations. The microscopic inspection of the fabricated MN arrays (Fig. 1) revealed that conical-shaped MN arrays (3 × 3) with sharp tips were formed that were around 780 ± 60 μm in height, 300 ± 40 μm in base width and 50 μm of interspacing between the MNs. However, higher polymer concentrations were required to fabricate MNs from low MW PVP hydrogels. The required polymer concentration used in the preparation of hydrogels from PVP K15, PVP K30 and PVP K29–32 were 60, 40 and 30 % *w*/*v*, respectively. Essentially, we were also able to encapsulate the three model drugs, with increasing MWs and at different concentrations within the MN arrays, while maintaining their mechanical properties (discussed later). However, it was noticed that using the same PVP hydrogels to form the MN baseplates produced very brittle and fragile MN baseplates. Therefore, a high MW PVP (i.e. PVP K90) hydrogel prepared at 15 % *w*/*v* was used to from a more a resilient and rigid baseplate for all the MNs tested in this study.

To ensure dosage accuracy and reproducibility, the drug content of MNs was determined (Table 2). Drug recovery from MNs was found to range from 92 ± 5 % to 97 ± 4 %. As shown in Table 2, the content of drug in the MN arrays (i.e. total amount present in nine needles) ranged from 0.96 ± 0.13 to 9.91 ± 0.28 μg for different MN formulations. Importantly, it is of great interest that we were able to incorporate approximately 1.3 and 10 μg of a high MW molecule (i.e. FD 70) in the PVP MNs when loaded at 2 and 10 mg/ml, respectively. A similar trend was noticed for other model drugs. Thus, dissolving MNs could be used to deliver clinically relevant doses of highly potent high MW anti-VEGF drugs such as Avastin® (bevacizumab, ≈149 kDa). For example, a study by Kim et al. [37] concluded that a dose of 4.4 μg of bevacizumab is needed to provide the same effect as that of 2.5 or 52.5 mg when delivered via the subconjunctival route or eye drops to treat CNV. Moreover, increasing the number of MN arrays can further increase drug content in the PVP MNs. However, it is important to remember that high density of MNs, unlike for transdermal application, can impair MN penetration into ocular tissues due to its curved surface or a bed of nail effect can occur.

**Table 2:** Drug and moisture content of MNs. Mean ± SD, *n* = 3

MN array code	Drug content (μg/array)	Moisture content (%)
F1	1.02 ± 0.18	7.64 ± 0.23
F2	1.31 ± 0.12	10.53 ± 1.03
F3	1.31 ± 0.17	10.26 ± 0.84
F4	9.91 ± 0.28	8.03 ± 0.23
F5	1.03 ± 0.18	9.89 ± 0.97
F6	0.96 ± 0.13	7.21 ± 0.65

Moisture content plays an important factor on mechanical properties of the MNs including rigidity, flexibility and dissolution kinetics within the ocular tissues that in turn effects drug stability and efficacy. High content of moisture will impair MNs ability to penetrate the tissues. In general, the moisture content of the MN arrays ranged from 7.21 ± 0.65 to 10.53 ± 1.03 %, which is dependent upon the PVP MW and type and concentration of the encapsulated drug, as shown in Table 2. Any level of moisture can affect the stability of drug molecules; however, this is particularly important for biologics that are high sensitive to moisture. Although these MN arrays were fabricated under normal lab conditions, fabrication of these MNs in a controlled environment (temperature and humidity) followed by low-temperature drying can further decrease the moisture content of MNs thereby enhancing the storage stability of biomolecules. Nevertheless, at these moisture levels, we found that the MNs maintained their mechanical strength and effectively penetrated the ocular tissues.

**Fig. 2:**
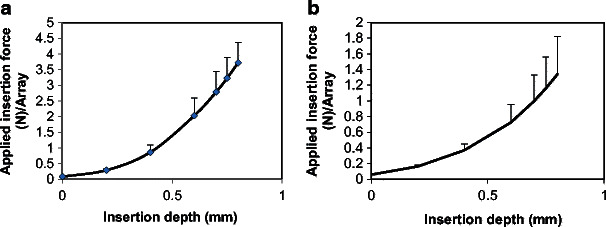
Graphical representation of the average depth of insertion vs forces applied for a 3 × 3 MN array (F2) into **a** the corneal and **b** the scleral tissues. Mean ± SD, *n* = 3

MN arrays were also tested for intraocular insertion forces. Prior to this, the isolated porcine corneal and scleral tissues were measured for thickness after hydration for 2 h. The average thickness was found to be 950 ± 70 and 700 ± 300 μm, for the cornea and sclera, respectively. Therefore, the MN height was chosen to be 800 μm to allow intrascleral or intracorneal drug delivery using MN arrays. Applying the MN arrays onto the tissues requires an appropriate force to enable MN penetration which should be maintained until MN array is completely dissolved to deliver its payload into the tissue. Literature review revealed no data has been reported concerning the required force for intraocular MN arrays insertion. Therefore, this study endeavoured to measure the required force (N) to insert MN arrays into either the corneal or scleral tissues in vitro. The applied insertion force/array vs MN insertion depth is reported in Fig. 2. In general, the fabricated MN arrays were sufficiently rigid to be inserted into the ocular tissues. In both ocular tissues, the required force to insert the 3 × 3 MN array was proportional to the insertion depth. This is can be attributed to the conical shape of MNs, where the MN diameter increase from tip to base thereby requiring higher forces with an increase in the depth of penetration. Interestingly, the required force to insert the MN array into the corneal tissue was higher than that for the scleral tissue. For example, the average insertion force required to fully insert the MN array into the corneal tissue was 3.72 N/9 MNs, which was nearly threefold higher than that required to be fully inserted into the scleral tissue (1.34 N/ 9MNs). Thus, the required insertion force per single MN is 0.41 and 0.15 N for the cornea and sclera, respectively.

In the literature, forces to penetrate various areas of the eye wall using hypodermic needle were reported [38, 39]. For example, Matthews et al. reported scleral penetration forces of around 1.0 N, when a single 18 G hypodermic needle with an outer diameter (OD) of 1.27 mm was used. However, the force required to penetrate the central cornea was significantly lower than all other areas i.e. around 0.5 N [38]. Although this contradicts our data, this study was conducted on the human eyeballs, unlike the porcine tissues reported in our study. Tissue type (porcine vs human) and intraocular pressure play a great role in defining the insertion forces. It is well documented that the cornea also has highly ordered parallel fibrils that make the tissue highly resistant to deformation at raised intraocular pressure. Upon the application of a tensile stress, a restoring force is generated by the stretched fibers that balances the applied force and resists deformation, providing the cornea with substantial tensile strength [40]. Thus, higher insertion force is required in the cornea compared to the sclera. But this can be explained on the basis that Matthews et al. have used hypodermic needles which differed from our study in relation to materials, dimensions and geometrics. Pulido et al. found peak needle penetration forces for the anterior sclera of 0.29 and 0.61 N with 30 G (OD 310 μm) and 27 G (OD 410 μm) needles, respectively [39]. The 30 G hypodermic needle is very closely related to our fabricated dissolving MNs in its OD, which is around 300 μm. However, the required insertion force was twofold higher than in our case. This can be attributed to the fact that our MNs are of conical shape while the hypodermic needles are of cylindrical shape. Thus, our MNs allow minimally invasive means of drug delivery.

**Fig. 3:**
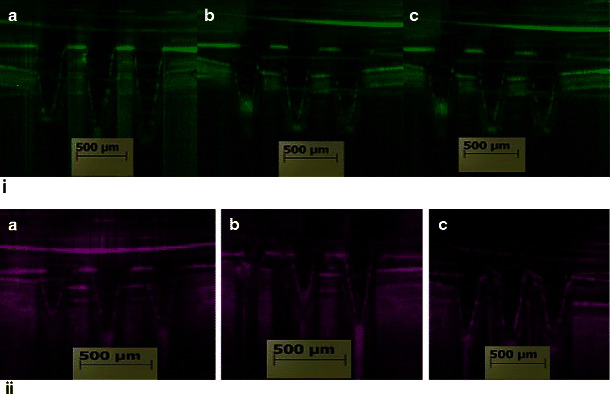
Sample OCT images of MN arrays inserted into a porcine (*i*) corneal and (*ii*) scleral tissue prepared from different PVP hydrogels namely (*a*) F2, (*b*) F5, and (*c*) F6

Further studies were performed to evaluate the ability of MN arrays to penetrate through the ocular tissues using OCT scanning. Images of MN arrays following their in vitro insertion into porcine corneal and scleral tissues are shown in Fig. 3. A previous report has shown that only 200 μm of the 650 μm height of a PVP-based 100-MN array was inserted into cadaver porcine skin [26]. Interestingly, the results here clearly demonstrate that all fabricated MN arrays from the three different PVP polymers i.e. PVP K15, PVP K30 and PVP K29–32 were strong enough to penetrate the ocular tissues in vitro without bending or breaking. The insertion depth was around 75 ± 10.8 % (*n* = 3) of the total MN height (data not presented). This can be attributed to that of our MN arrays as they are composed of fewer MNs (only nine MNs) with different geometrics, and the use of ocular tissues instead of skin, which has differed mechanical properties. The partial insertion of MNs can be ascribed to the use of the highly elastic Parafilm® layer (to avoid dissolution of MNs during imaging) hindering the MN arrays from being fully inserted into the ocular tissue. However, this should not be of great concern due to the fact that in real situations, MN arrays will be applied directly into the ocular tissues without any barrier. Thus, high percentage (>75 %) of MN insertion will be expected.

**Fig. 4:**
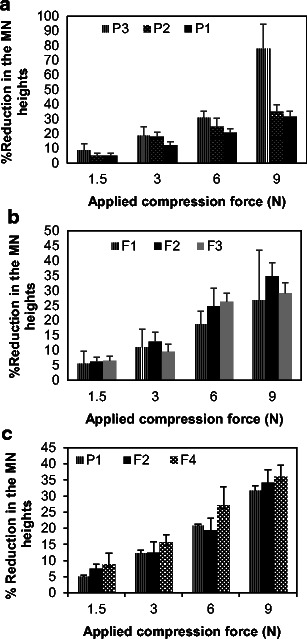
Graphs representing the average percent reduction in MN height vs predefined compression forces. MN arrays were fabricated either from **a** plain PVP hydrogels i.e. P1, P2 and P3, **b** drug-loaded PVP MNs i.e. F1, F2 and F3 (at a concentration of 2 mg/ml). **c** PVP K29/32-based MN arrays encapsulating the model drug FD 70 at a concentration of either 0 (P1), 2 (F2) or 10 mg/ml (F4). Data reported as mean ± SD, *n* = 3

**Fig. 5:**
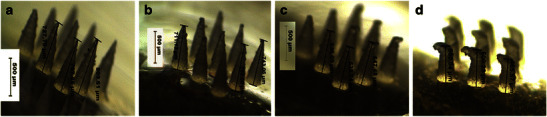
Sample images of PVP K29/32-based MN arrays encapsulating FD 70 at a concentration of 2 mg/ml, before and after applying predefined compression forces (in N), the applied forces are **a** 0 N/array, **b** 1.5 N/array, **c** 3 N/array and **d** 9 N/array

The ability of dissolving MN arrays to withstand the forces required for insertion into the designated biological tissue or any additional accidental forces while inserting is the key factor in their successful development and application. Mechanical strength of the dissolving MN arrays is widely affected by several factors including polymer type and concentration, moisture content, encapsulated drug type and concentration and preparation methods [26, 41]. Therefore, in this study, we evaluated the mechanical strength (fracture force) of those MN arrays prepared from either both plain PVP and PVP/drug hydrogel formulations. A Texture Analyser (in compression mode) was used to apply predefined forces including those required for MN insertion and other higher forces (1.5, 3, 6 and 9 N). The percentage of MN height reduction was calculated and plotted against the applied compression force and is presented in Fig. 4. Images of some samples MN arrays after applying predefined forces are presented in Fig. 5.

As represented in Figs. 4 and 5, PVP polymers at the specified hydrogel concentrations can form sufficiently rigid but brittle MN arrays. PVP K15 produced the most brittle MN arrays that showed around 18 % reduction in the MN height at a force of 3 N/array, but PVP K30 and PVP K29/32 MNs showed around 12.9 and 12.4 % reduction in height when exposed to the same force. Additionally, data shows that there is a proportional relationship between the applied compression force and the percentage of reduction in the MN height, and this is more profound in MN arrays fabricated from PVP K15. PVP K29/32 (P1) formed the most rigid and least brittle MN arrays, which can withstand high forces with minimal height reduction. For example, applying forces between 1.5 and 9 N/array caused an average height reduction from 5 to 32 %. PVP K30 (P2) comes second in MN rigidity. However, no significant (*p* > 0.05) difference in the mechanical strength (fracture force) was noted between MN arrays when fabricated from PVP K30 or PVP K29/32 hydrogels. This can be ascribed to that both polymers have an intermediate and closely related MW i.e. 40 k and 58 k Da, respectively.

Interestingly, encapsulated model drugs of different MWs at a concentration of 2 mg/ml in hydrogel did not significantly affect the mechanical properties when low compression forces were applied such as 1.5 and 3 N/array. However, the effect was more profound with high compression forces such as 6 or 9 N/array. But statistically no significant difference (*p* > 0.05) was found among the three model drugs in terms of the effect on the mechanical strength within the same MNs and at same concentration (Figs. 4b and 5). On the other hand, increasing the encapsulated drug from 0 to 10 mg/ml in PVP K29/32-based MN arrays produced a gradual decrease in the mechanical strength of the MNs. For example, applying 3 N/array on MN arrays containing no drug, FD 70 at 2 mg/ml or FD 70 at 10 mg/ml, caused a height reduction of around 12.4 ± 2.5, 12.4 ± 3.5 and 15.7 ± 2.3 %, respectively. This can be attributed to a possible interaction between the FD 70 and the PVP polymer which may affect the polymer configuration upon recrystallization during the drying process. Our PVP K29/32-based MNs were still significantly stronger than other PVP-based MNs reported in literature, where Sullivan et al. found the fracture force of MNs to be 0.13 ± 0.03 N per needle [26]. This might be due to the method of MN fabrication which employed the polymerization technique.

**Fig. 6:**
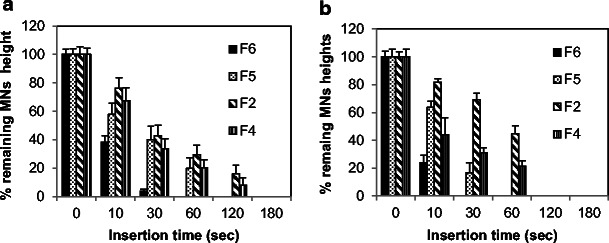
Percentage of remaining MNs heights vs dissolution time of PVP-based MN arrays encapsulating the model drug FD 70 after insertion for predefined times into porcine ocular tissues; **a** the corneal tissues, **b** the scleral tissues. MN arrays were fabricated using various PVP/drug hydrogel formulations: PVP K29/32/ FD70 (F2); PVP K29/32/ FD70 (F4); PVP K30/ FD70 (F5); PVP K15/ FD70 (F6). Data reported as mean ± SD, *n* = 3

**Fig. 7:**
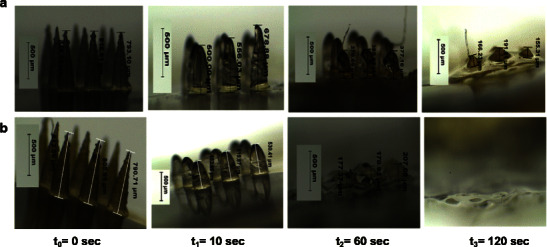
Representative digital images of PVP K29/32-based MN arrays encapsulating FD 70 at a concentration of 2 mg/ml before and after insertion into porcine **a** the corneal and **b** the scleral tissues for predefined times; *t*_0_ = 0 s, *t*_1_ = 10 s, *t*_2_ = 60 s, *t*_3_ = 120 s

Understanding the behavior of MN after insertion into the ocular tissue is essential in the design and development of an effective MN array and to determine optimal conditions in terms of the MN application method. Therefore, the dissolution kinetics of the PVP-based MN arrays was evaluated following insertion into the porcine ocular tissues, as shown in Fig. 6. Although, the water content of porcine corneal tissue (around 78 %) is higher than sclera tissue (around 71 %) [28, 42, 43], our data showed that the PVP-based MN arrays dissolved faster in the scleral tissues (Figs. 6 and 7b) than in the cornea (Figs. 6 and 7a). This could be due to the lipoidal nature of the corneal epithelium, and it is also depended upon the degree of hydration of the tissues prior to experimental setup. With regard to the effect of PVP’s MW on the dissolution rate, results indicate MNs fabricated from PVP K15 were completely dissolved within 30 s following insertion into the sclera and less than 60 s in the cornea. Interestingly, within the first 10 s of insertion, around 80 and 60 % of the PVP K15-based MNs was dissolved within the sclera and cornea. PVP K30-based MN arrays dissolved in similar fashion but at slower rate in comparison with those MN arrays fabricated from PVP K15. In contrast, PVP K29/32-based MN arrays completely dissolved within 120 and 180 s following insertion into the porcine scleral and corneal tissues, respectively. Therefore, given the similarity of porcine and human ocular tissues, we expect that our MN dissolution in the human eye could also be occur within similar magnitude.

Our data revealed that PVP-based MNs dissolve faster in ocular tissues than in the skin. This was expected in comparison to the data in literature, if we take into account that the ocular tissues vary in their properties and water content compared to the skin [44]. In fact, it was reported that after insertion into cadaver porcine skin, PVP-based dissolving MNs showed significant dissolution within 1 min, and after 5 min, the MNs were 89 % (by mass) dissolved [25]. With regard to the effect of the encapsulated drug type and concentration on the dissolution behaviour of the fabricated MN arrays, results (not presented) indicated that drug MW at low concentrations (2 mg/ml) had no significant effect. However, increasing FD 70 concentration from 2 to 10 mg/ml resulted in faster dissolving MN arrays. Overall, results revealed that PVP K29/32 MNs provide rapid dissolution in ocular tissues, which make them as a possible candidate in the fabrication of MN arrays for ocular application. However, using PVP K15 MNs are not feasible as MN arrays could dissolve very quickly even before complete insertion into the ocular tissues thereby depositing its payload on the ocular tissue surface instead within the tissue.

### In vitro intraocular drug distribution studies

**Fig. 8:**
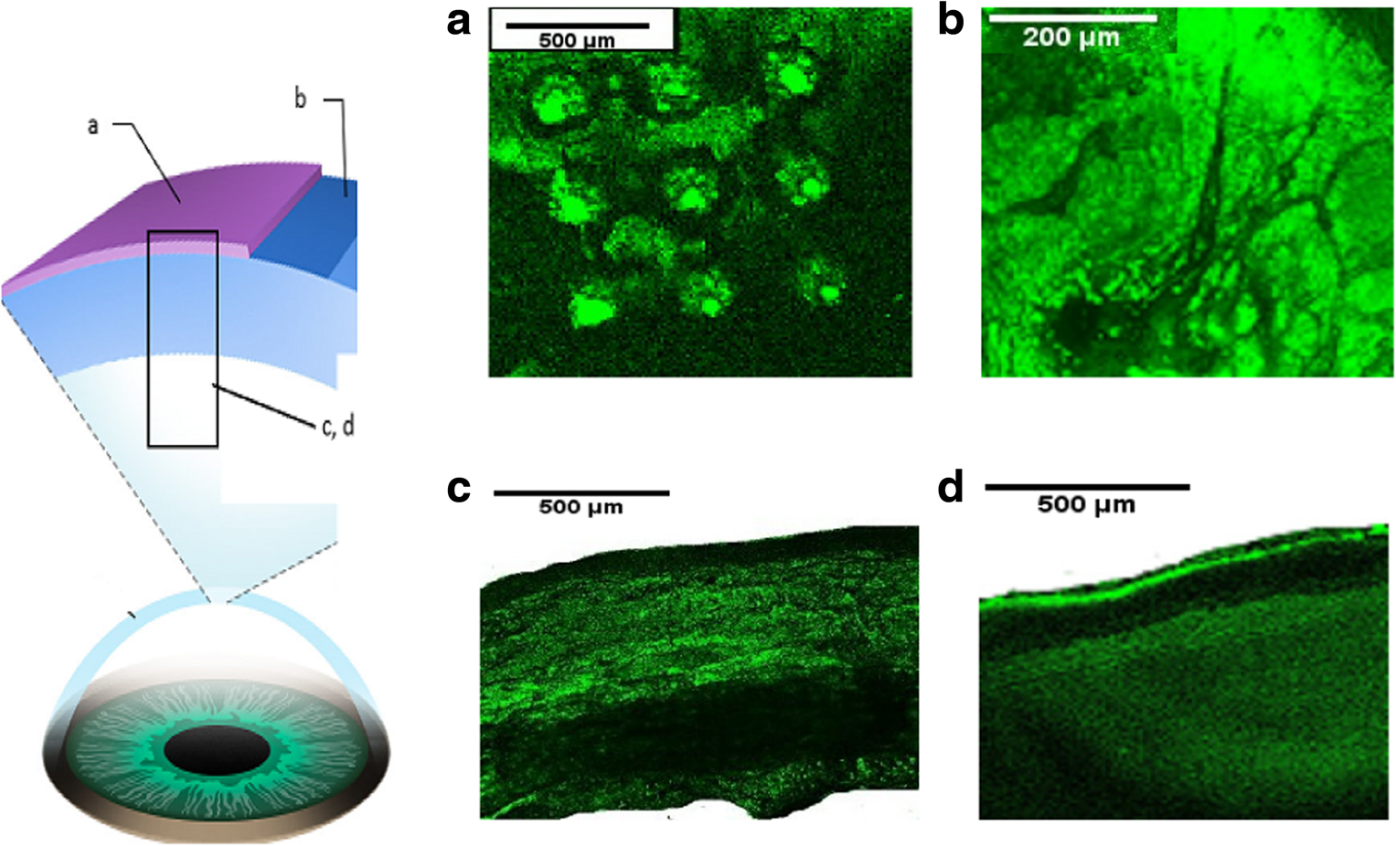
Schematic diagram on the *left hand side* represents the collection and processing of confocal images of the corneal tissue following application of FD70-loaded PVP K29/32 MN arrays (F2), where confocal images represents **a** after 5 min of MN application, **b** topical image at a depth of 80 μm from surface of the tissue after 1 h following MN array insertion, **c** cross-section image of tissue after 1 h following MN array insertion and **d** cross-section image of tissue after 1 h applying an aqueous solution of FD 70 (at 2 mg/ml)

**Fig. 9:**
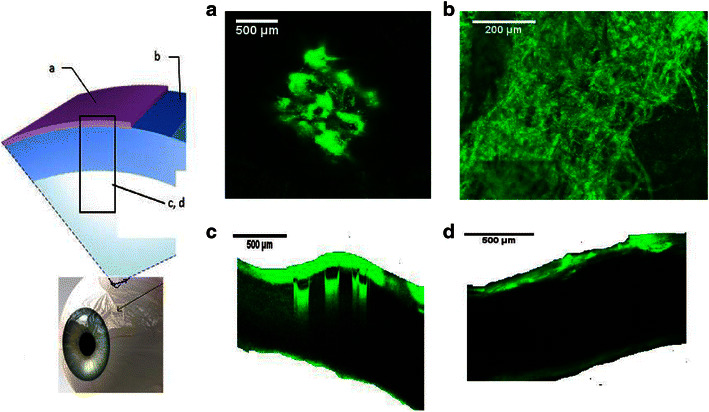
Schematic diagram on the *left hand side* represents the collection and processing of confocal images of scleral tissues following application of FD70-loaded PVP K29/32 MN arrays (F2), where **a** topical image of tissue after 5 min following insertion of MN array, **b** cross-section image of tissue after 5 min following MN array insertion, **c** topical image at a depth of 80 μm from surface of the tissue after 1 h following MN insertion and **d** cross section image of tissue 1 h after applying an aqueous solution of FD 70 (at 2 mg/ml)

**Fig. 10:**
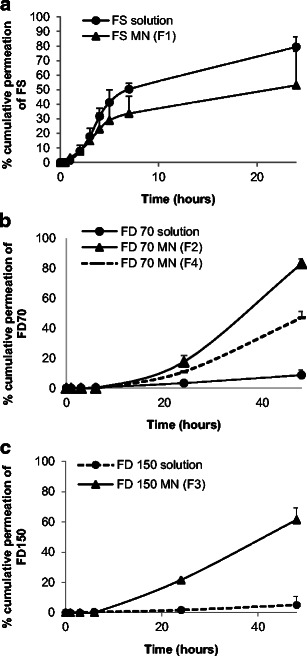
Graphical representation of in vitro permeation profiles through the corneal tissue from either aqueous solution or PVP K29/32-based MN arrays of three model drugs namely **a** FS, **b** FD 70 and **c** FD 150

In addition to insertion of MNs into the tissues, it is also essential to get insight into drug distribution within the ocular tissue after application. Therefore, in this study, the fluorescently conjugated model drugs were traced within the ocular tissue using scanning confocal microscopy, as shown in Figs. 9 and 10. Results showed that MN arrays fabricated from PVP K29/32 polymer were able to penetrate both the porcine sclera and cornea. Although, the MN heights are around 800 μm, they did not completely pierce either ocular tissue. This can be seen clearly in Figs. 8c and 9c. It suggests that dissolving MNs start dissolving as soon as they are inserted into the ocular tissues and do not pierce it completely (as proven in OCT studies). This is consistent with our findings from dissolution studies in the previous section where we found that more than 20 and 40 % of the MNs dissolved within 10 s after insertion either in the cornea or in the sclera. This could be of great benefit, as there will be no concerns of causing damage to the innermost sensitive ocular tissues such as the choroid/retina beneath the scleral tissue. Furthermore, the confocal images show that macromolecules such as FD 70 cannot permeate easily through the scleral or corneal tissues. This can be clearly concluded from images of the corneal and scleral tissues after 1 h from application of FD 70 aqueous solution (Figs. 8d and 9d). Our findings completely agree with data reported in literature that the cornea and sclera form a strong barrier to molecules larger than 5 k Da. Therefore, large molecules such as FD 70 are more likely to accumulate in the outer surface of the sclera and cornea, when they are applied topically [45–47]. Essentially, as we can see in Fig. 9a, dissolving MNs were able to penetrate the epithelium (outer layer of the cornea with thickness of around 50 μm), which is deemed the main barrier for transcorneal drug permeation and then reached the stroma and dissolved to release the payload that led to formation of drug depot (Fig. 8b, c). Similarly, dissolving MNs were able to penetrate the scleral tissue (Fig. 9a, b) to form a drug depot within the tissue that diffused to the other side of the sclera (Fig. 9c).

In terms of the small MW hydrophilic model drugs (i.e. FS), MN arrays successfully delivered the drug into the ocular tissues (data not shown), which then distributed evenly within the tissue. However, no significant difference was seen in distribution of FS within the tissues from either inserting MNs containing FS or application of FS aqueous solution topically. Our finding are in agreement with data reported in literature that FS, a water-soluble compound with a MW of 376 Da, penetrates into and permeates across both the corneal epithelial and scleral membrane [45–47]. Thus, using dissolving MN arrays to deliver such hydrophilic drugs with small MW do not offer great advantages in comparison to topical drops, apart from increasing their retention time in the ocular tissue. Accordingly, dissolving MN arrays could be useful in facilitating delivery of macromolecules by two mechanisms. For macromolecules, by bypassing the main barrier for their permeation either across the epithelium in cornea or in the sclera and reducing the diffusion time through the ocular tissues and by increasing their retention time in the tissue thereby preventing their removal by blinking and tear secretion upon topical application. And, for small molecules, MNs can increase their retention time in the ocular tissues.

### In vitro permeation studies

**Fig. 11:**
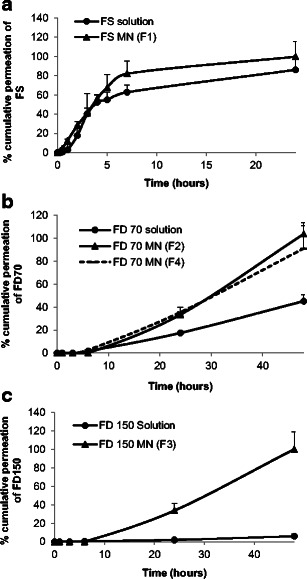
Graphical representation of in vitro permeation profiles through the scleral tissue from either aqueous solution or PVP K29/32-based MN arrays of three model drugs namely **a** FS, **b** FD 70 and **c** FD 150

Taking into consideration the properties of rapidly dissolving MN arrays fabricated, from the three PVP polymers i.e. PVP K15, PVP K30 and PVP K29/32, we have selected PVP K29/32 MNs to investigate the efficiency for ocular drug delivery via intrastromal and intrascleral application. Drug permeation studies were performed using either these MN arrays or predefined volume of the respective drug aqueous solutions. Data concerning drug permeation profiles through the cornea and sclera are presented in Figs. 10 and 11, respectively.

For the small MW molecule (i.e. FS), a high permeation (Figs. 10a and 11a) was seen across the corneal and scleral tissues. The percentage permeation across the sclera was nearly the same from either topically applied aqueous solution or following MN application. For example, 86 and 99 % of FS permeated after 24 h from MNs or solution, respectively. In contrast, the percentage permeation across the cornea was significantly higher for the topically applied aqueous solution (79 %) when compared to MNs (53 %). In general, the drug first permeated and increased at a constant rate until 6 h, after which the drug permeation rate decreased significantly. This could be due to FS binding strongly to the scleral and corneal tissues. The binding was likely to be mediated through ionic interactions since fluorescein has three dissociation constants that correspond to its three pK_a_ values: 2.13, 4.44 and 6.36, respectively. At pH 7.4, the anionic forms of FS may have bound to positively charged extracellular matrix molecules (e.g. collagens) and proteins dissolved in interstitial fluid. The binding may effectively reduce the flux of FS in tissues. Our observations are consistent with other literature data [47]. Furthermore, FS diffusion through the corneal tissue from inserted MNs was slower than the topically applied drug aqueous solution. This suggests that PVP polymer from dissolved MNs may encapsulate the drug reducing its diffusion from stroma to the endothelium thus permeation into the receptor solution thereby showing a sustained release profile.

For macromolecules such as FD 70 and FD 150, topically applied aqueous solutions showed high resistance in permeation across both the scleral and corneal tissues (Figs. 10 and 11). Though FD 70 showed a high permeation profile through both the cornea and sclera when compared to FD 150. For example, the average percentage permeation of aqueous solutions of FD70 and FD150 was 9 and 5 % across the corneal tissues. In contrast, average percentage permeation of aqueous solutions of FD 70 and FD 150 was 45 and 6 % across the scleral tissues. Concerning the effect of MNs in enhancing the transocular permeation of both macromolecules, our results indicate that dissolving MN arrays have significantly enhanced transocular delivery of both model macromolecules in comparison with aqueous solutions. The percentage drug delivered through the cornea for FD 70 from topically applied aqueous solutions was 9 % which increased to 83 % after 48 h. Likewise, permeation of FD 150 increased from 5 to 61 %. This suggests that dissolving MN arrays played a significant role in enhancing delivery into the corneal stroma and formed a depot, but the endothelium is still forming a strong barrier. Thus, the drug MW still plays its role in dictating transcorneal permeation. However, intrastromal delivery can be an excellent target in the treatment of corneal diseases such as CNV. On the other hand, increasing the drug concentration of FD 70 in the dissolving MN arrays by fivefold (2 to 10 mg/ml) did not significantly increase the percentage permeation. In the sclera, dissolving MNs improved delivery of both small and macromolecule drugs. For the macromolecules, i.e. FD 70 and FD 150, dissolving MNs successfully delivered the total applied doses from both molecules.

### Biocompatibility

**Fig. 12:**
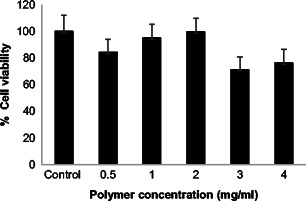
Graphical representation of percent retinal cell counts in cell cultures impregnated with predefined amounts of PVP K29/32 in cell culture media of a 0.5, 1, 2, 3 and 4 mg/ml

PVP polymer is considered to be biologically inert. In this study, we investigated the biocompatibility of PVP polymer with ARPE cells. Cells were exposed to a range of polymer concentrations (at 0.5, 1, 2, 3 and 4 mg/ml) to mimic a scenario of retinal cells-polymer exposure upon delivering the polymer in the scleral tissue. The percentage viability was found to be >83 % at all concentrations below 2 mg/ml (Fig. 12), which concludes that PVP K29/32 MNs is non-toxic in an in vitro testing to the retinal cells, therefore, deemed as biocompatible. However, cell viability was significantly inhibited when cells were treated with 3 (*p* = 0.031) and 4 mg/ml (*p* = 0.041) of PVP. This could be primarily due to excess amount of material that could have inhibited normal cell growth.

## Conclusion

This study, for the first time, demonstrated the design and fabrication of rapidly dissolving MN arrays to overcome the barrier function of ocular tissues in order to improve ocular drug delivery. In doing so, a simple and cost-effective mould-casting method was developed that utilizes the relatively economic biocompatible polymer, PVP, in MN fabrication. Of the three PVP MWs (i.e. PVP K15, PVP K30 and PVP K29/32) investigated, PVP K29/32-based MNs were able to withstand the higher compression force required for insertion in the ocular tissues with minimal reduction in needle height without fracturing or bending and dissolved in the ocular tissues within less than 3 min. These MNs were able to encapsulate either one of three model drugs of various MWs ranging from 376 to 150,000 Da. In vitro studies showed that MNs penetrated into the ocular tissues could rapidly dissolve to form a depot within the tissues and showed a higher degree of permeation of high MW model molecules and sustained their release. This can be considered of great importance for delivery of high MW anti-VEGF drugs in a minimally invasive manner. Additionally, the material used in the fabrication of MNs was found to be biocompatible to retinal cells. Because MNs dissolve in the ocular tissue fluid, there is no sharps waste, which makes dissolving MNs impossible to reuse and thereby eliminates the risks of biohazard sharps, unlike intravitreal injections that use hypodermic needles. Further studies are required to investigate the feasibility of using this platform to deliver active therapeutic proteins such bevacizumab, ranibizumab or aflibercept which are used in the treatment of posterior (AMD) and anterior (CNV) ocular diseases.
