# Correction to: Rapidly dissolving polymeric microneedles for minimally invasive intraocular drug delivery

**DOI:** 10.1007/s13346-026-02073-1

**Published:** 2026-02-20

**Authors:** Raghu Raj Singh Thakur, Ismaiel A. Tekko, Farhan Al-Shammari, Ahlam A. Ali, Helen McCarthy, Ryan F. Donnelly

**Affiliations:** 1 School of Pharmacy, Medical Biology Centre, Queen’s University Belfast, 97 Lisburn Road, Belfast, BT9 7BL Northern Ireland UK; 2 Faculty of Pharmacy, Aleppo University, Aleppo, Syria

**Correction to: Drug Deliv. and Transl. Res. (2016) 6:800–815**

https://doi.org/10.1007/s13346-016-0332-9

In the original online version of this article, the OCT image shown in Fig. 3(i)(c) (formulation F6) was inadvertently duplicated from the adjacent panel.

To address this, the authors reproduced the F6 OCT experiment using the same formulation, materials, and OCT imaging setup. The updated Fig. 3 has been prepared with the corrected panel (i)(c), while all other panels remained unchanged. The updated image does not change any results, data interpretation, or conclusions of the paper, and there is no alteration to the scientific content of the article.

Following is the correct Fig. 3:

**Fig. 3:**
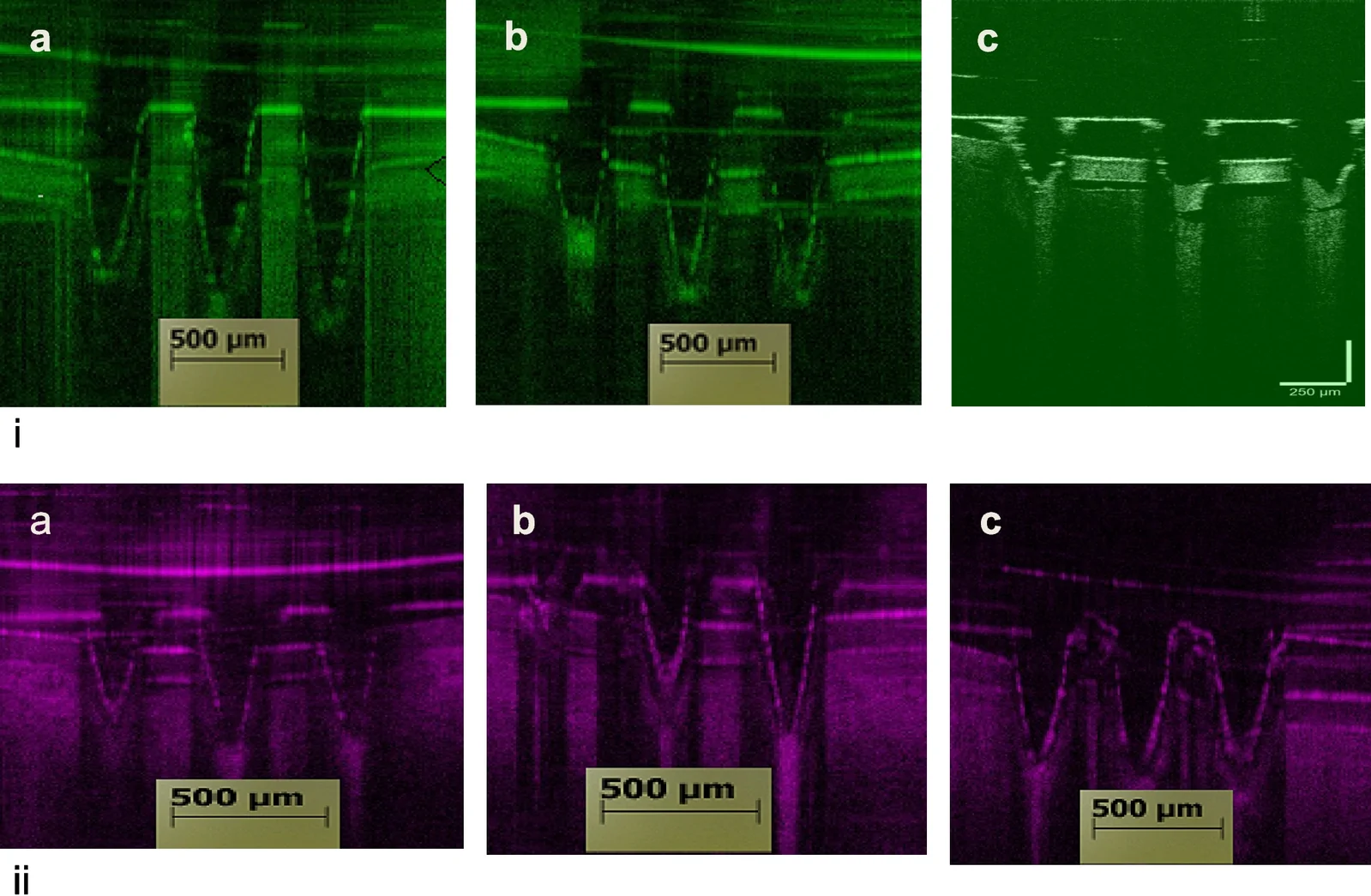
Sample OCT images of MN arrays inserted into a porcine (**i**) corneal and (**ii**) scleral tissue prepared from different PVP hydrogels namely (**a**) F2 (**b**) F5 and (**c**) F6

